# Determinants of Negative Childbirth Experience and Its Influence on Mode of Delivery Decisions

**DOI:** 10.7759/cureus.92825

**Published:** 2025-09-21

**Authors:** Sana Danish, Asma Ambareen, Shama Chaudhry, Maryum Sana

**Affiliations:** 1 Obstetrics and Gynaecology, Shaikh Zayed Hospital, Lahore, PAK; 2 Obstetrics and Gynaecology, Khyber Teaching Hospital, Peshawar, PAK; 3 Obstetrics and Gynaecology, Dr. Ziauddin University, Karachi, PAK; 4 Nursing and Patient Care, University of Health Sciences, Lahore, PAK

**Keywords:** cesarean section, delivery, obstetric, obstetric labor complications, parturition, postpartum period

## Abstract

Background

Maternal perception of childbirth plays a major role in postpartum psychological well-being and subsequent birth choices. Emotional distress, interruption of maternal-infant bonding, and reluctance toward future pregnancies may arise as a result of negative experiences during delivery. This study aimed to explore the socio-demographic, obstetric, and psychosocial factors related to adverse or positive birth experiences and their influence on shaping future preferences in childbirth.

Methods

An observational cross-sectional study was conducted on 72 postpartum women admitted to a tertiary care hospital. Participants were categorized into two groups based on their self-reported childbirth experiences: negative (n = 48 [0.67%]) and positive (n = 24 [0.33%]) groups. Structured interviews were used to collect data in the first 48 hours. The data was collected on variables including maternal age, education level, parity, mode of delivery, labor length, use of pain relief, presence of a birth companion, and personnel conduct. Chi-square and t-tests were used to perform statistical analysis with a significance level of p < 0.05.

Results

Negative experiences were significantly associated with emergency cesarean sections (18 [37.5%] against 3 [12.5%], p = 0.038), labor lasting more than 12 hours (30 [62.5%] vs. 7 [29.2%], p = 0.011), and failure of pain relief measures (29 [60.4%] vs. 4 [16.7%], p = 0.001). The absence of a birth companion (10 [20.8%] vs. 17 [70.8%], p < 0.001) and poor staff attitude (30 [62.5%] vs. 3 [12.5%], p < 0.001) were also associated with the negative experiences. A total of 20 (83.3%) women in the positive group felt supported by professionals, as compared to 20 (41.7%) women in the negative group. The approximate levels were the same with perceived safety (21 [87.5%] vs. 26 [54.2%]) and participation (17 [70.8%] vs. 22 [45.8%]). Women with higher education and urban residency had better chances of reporting positive experiences.

Conclusion

The modifiable factors, including clinical interventions, pain relief, labor management, support systems, and staff conduct, have a strong impact on negative childbirth experiences. Increased childbirth satisfaction and the determinant of future birth delivery can be greatly improved using better maternity care, adequate pain relief, and support during the labor process.

## Introduction

Childbirth is one of the most crucial life experiences that impacts the long-term psychological and emotional well-being of a woman [1]. Although childbirth has been termed empowering by most women, others have experienced negative outcomes [2]. Recent literature indicates that approximately one-third of women worldwide have reported an adverse childbirth experience [3]. These experiences often lead to postpartum depression, trauma, difficulties with the maternal-infant relationship, and pregnancy avoidance in the future [4].

Childbirth perception is affected by several obstetric, psychosocial, and interpersonal factors [5]. Studies indicate that not only clinical strategies but also the emotional environment surrounding labor are crucial factors contributing to maternal perceptions and overall satisfaction [6]. Moreover, negative perceptions are also commonly linked to clinical events such as emergency cesarean section and long labor [7]. Similarly, insufficient pain management, absence of a birth companion, and negative interactions with the staff may adversely influence the emotional responses in women [8]. Although these associations have been previously studied, the cumulative effect on long-term outcomes of health processes, including emotional recovery, personal well-being, and future delivery preferences, remains under-researched. Therefore, understanding the mutual influence of interconnected factors on maternal health outcomes becomes imperative. This knowledge can inform patient-centered maternity care and inform policies to improve psychological recovery and positive childbirth experiences. This study hypothesized that childbirth experiences are affected by modifiable factors, including support, provider attitudes, and birth-related habits.

The objective of this study is to determine the combined effect of socio-demographic, obstetric, and psycho-social factors on childbirth perception. This study also explores how these experiences influence future decisions regarding the mode of delivery that can enhance maternal satisfaction and psychological recovery.

## Materials and methods

The cross-sectional design of this study was employed to investigate the determinants of poor childbirth outcomes and their relationship with positive and negative childbirth experiences at a University of the Punjab-affiliated tertiary care facility (Ref: 143/10/2023) from October 2023 to April 2024, following informed consent. A consecutive non-probability sampling method was used to recruit women in the maternity ward after birth. Participants were approached within 48 hours after delivery and gave informed consent before enrollment. The study sample consisted of 72 postpartum women. The sample size was determined by OpenEPI version 3.0.0 (released 2013, Atlanta, GA, USA). Women eligible for inclusion were aged 18 years or older, had a singleton live birth, and were able to communicate in the local language. Participants were excluded if they had a stillbirth, severe postpartum complications, or a psychiatric disorder or refused to participate.

The study population was divided into two groups: negative (n = 48, 67%) and positive (n = 24, 33%) according to their self-reported experience of childbirth. Rather than active intervention measures, natural exposures were studied. Study variables included socio-demographic characteristics (age, education, employment, residence), obstetric characteristics (parity, duration of labor, mode of delivery), and psychosocial/hospital factors (pain relief, existence of birth companion, staff attitude). Procedural adherence to exposures was not externally regulated.

A structured interview was utilized by trained personnel to collect data. The authors of the study developed a self-developed questionnaire (English version) to ask the patients. The questionnaire included socio-demographic, obstetric, psychosocial, and childbirth-related questions. The complete questionnaire is provided in Appendix. IBM Corp. Released 2017. IBM SPSS Statistics for Windows, Version 26.0. Armonk, NY: IBM Corp. was used in statistical analysis. Categorical variables were subjected to chi-square tests, and continuous variables to independent t-tests. A statistically significant p-value was set at < 0.05.

## Results

Participants consisted of 72 women with childbirth experience, 48 (67%) of whom experienced negative experiences, and 24 (33%) reported positive experiences. Clinically, a negative experience was linked with increased emergency cesarean section and excessive labor. Psychosocially, less pain relief, the presence of the birth companion, and the helpful attitude of staff were lower in women in the negative group, indicating that the hospital environment includes changeable factors that can have a powerful impact on maternal satisfaction and subsequently the mode of delivery preferences. The socio-demographic determinants of childbirth experience are summarized in Table 1.

**Table 1:** Socio-Demographic Determinants of Childbirth Experience (n = 72) n: Number of participants, %: percentage, SD: standard deviation, *: Significant at p < 0.05, ”Credits: Sana Danish, Shama Chaudhary, Asma Ambareen, Maryum Sana”

Variable	Negative (n = 48)	Positive (n = 24)	Test Used	Test Value	p-value
Mean Age (years) (Mean ± SD)	27.6 ± 4.8	28.4 ± 5.1	t-test	t = -0.698	0.488
Education: Secondary or less (%)	33 (68.8%)	10 (41.7%)	Chi-square	χ² = 5.03	0.026*
Employed (%)	19 (39.6%)	14 (58.3%)	Chi-square	χ² = 2.29	0.130
Urban Residence (%)	29 (60.4%)	21 (87.5%)	Chi-square	χ² = 5.88	0.015*

A total of 33 (68.8%) women with negative experiences had secondary or less education compared to 10 (41.7%) in the positive experience group (p=0.026). Furthermore, the percentage of women residing in urban areas was significantly different between the negative (29, 60.4%) and the positive (21, 87.5%) groups (p = 0.015), suggesting that both the level of education and residence can affect the experience of childbirth satisfaction. Obstetric factors influencing birth experience are presented in Table 2.

**Table 2:** Obstetric Factors Influencing Birth Experience (n = 72) n: Number of participants, %: percentage, h: hours, *: Significant at p < 0.05, ”Credits: Sana Danish, Shama Chaudhary, Asma Ambareen, Maryum Sana”

Variable	Negative (n = 48)	Positive (n = 24)	Test Used	Test value	p-value
Primiparity (%)	32 (66.7%)	12 (50.0%)	Chi-square	χ² = 1.85	0.174
Spontaneous Vaginal Delivery (%)	22 (45.8%)	18 (75.0%)	Chi-square	χ² = 5.34	0.020*
Emergency C-Section (%)	18 (37.5%)	3 (12.5%)	Chi-square	χ² = 4.31	0.038*
Labor Duration >12h (%)	30 (62.5%)	7 (29.2%)	Chi-square	χ² = 6.44	0.011*

Obstetric factors showed significant differences. The rate of emergency cesarean section among women with negative experiences was 18 (37.5%) compared to 3 (12.5%) in the positive group (p = 0.038), and 30 (62.5%) had labor lasting more than 12 hours, compared with 7 (29.2%) of the positive group (p = 0.011). There was also a low percentage of spontaneous vaginal deliveries, with 22 (45.8%) as compared to 18 (75.0%) in the positive group (p = 0.020), which demonstrates the effect of complications on birth perception. Table 3 outlines the psychosocial and hospital factors of the childbirth experience.

**Table 3:** Psychosocial and Hospital Factors in Childbirth Experience (n = 72) n: Number of participants, %: percentage, SD: standard deviation, *: Significant at p < 0.05, ”Credits: Sana Danish, Shama Chaudhary, Asma Ambareen, Maryum Sana”

Variable	Negative (n = 48)	Positive (n = 24)	Test Used	Test value	p-value
Presence of Birth Companion (%)	10 (20.8%)	17 (70.8%)	Chi-square	χ² = 14.73	<0.001*
Received Pain Relief (%)	19 (39.6%)	20 (83.3%)	Chi-square	χ² = 11.15	0.001*
Staff Attitude Rated as Good (%)	18 (37.5%)	21 (87.5%)	Chi-square	χ² = 18.00	<0.001*
Mean Hospital Stay (days) (Mean ± SD)	3.6 ± 1.2	2.7 ± 0.9	t-test	χ² = 3.17	0.002*

The hospital-related and psychosocial variables varied significantly between the two groups. Having a birth companion present was associated with 10 (20.8%) of the women with negative experiences, as opposed to 17 (70.8%) of the positive group (p < 0.001). Only 19 (39.6%) of the women in the negative group received pain relief compared to 20 (83.3%) in the positive group (p = 0.001). Furthermore, the staff behavior was rated poorly by 30 (62.5%) of women in the negative group, whereas only 3 (12.5%) in the positive group gave a poor staff rating (p < 0.001). The average length of stay in hospital was also significant in the negative group (3.6 days compared to 2.7 days, p = 0.002). Overall, participants’ responses to the key experience domains are given in Table 4.

**Table 4:** Participant Responses to Childbirth Experience Domains (N=72) n: Number of participants; %: Percentage; SD: Standard deviation. Percentages are based on N: 72. For 'Negative Experience Determinants', 'Agree' indicates agreement with negative statements

Domain	No. of Items	Disagree/Strongly Disagree n (%)	Neutral n (%)	Agree/Strongly Agree n (%)	Mean ± SD
Own Capacity (Q7–Q9)	3	10 (13.9%)	12 (16.7%)	50 (69.4%)	3.28 ± 1.33
Professional Support (Q10–Q12)	3	9 (12.5%)	11 (15.3%)	52 (72.2%)	3.51 ± 1.30
Perceived Safety (Q13–Q15)	3	7 (9.7%)	12 (16.7%)	53 (73.6%)	3.61 ± 1.25
Participation (Q16–Q18)	3	12 (16.7%)	12 (16.7%)	48 (66.7%)	3.18 ± 1.36
Negative Experience Determinants (Q19–Q23)	5	19 (26.4%)	21 (29.2%)	32 (44.4%)	3.22 ± 1.37
Future Delivery Decision (Q24–Q28)	5	16 (22.2%)	19 (26.4%)	37 (51.4%)	3.38 ± 1.33

Domain-level responses were cross-examined to study how the objective factors influenced maternal perceptions. Despite the fact that average scores were positive, there were some obvious gaps. For example, 20 (83.3%) women in the positive group said that they felt supported by professionals, as compared to 20 (41.7%) women in the negative group. The approximate levels were the same with perceived safety (21, 87.5% vs. 26, 54.2%) and participation (17, 70.8% vs. 22, 45.8%). Women in the negative group, on the other hand, were observed to have a higher rate of endorsing items that showed negative experience determinants (32 [66.7%] vs. 6 [25.0%]). The influence of birth experience on future preferences for delivery is shown in Table 5.

**Table 5:** Preferred Mode of Next Delivery by Type of Birth Experience n: Number of participants; %: Percentage

Response	Negative Experience (n=48)	Positive Experience (n=24)	Total (N=72)
Vaginal Delivery	18 (37.5%)	20 (83.3%)	38 (52.8%)
Cesarean Delivery	20 (41.7%)	2 (8.3%)	22 (30.6%)
Undecided	10 (20.8%)	2 (8.3%)	12 (16.7%)

These varying perceptions had significantly contributed to subsequent childbirth intentions. Women with negative experiences were more likely to prefer cesarean section (20, 41.7%) or to remain undecided (10, 20.8%), whereas those with positive experiences favored a natural mode of delivery in the future (20, 83.3%).

## Discussion

The purpose of this study was to find out the socio-demographic, obstetric, and psychosocial determinants of negative childbirth experiences and to assess their impact on the preferences of pregnant women in future deliveries. The results validate the hypothesis that clinical conditions and interpersonal aspects of care highly contribute to maternal birth experiences.

The results of the socio-demographic factors showed that women with less education and women living in rural settings had greater negative experiences of childbirth. This observation is consistent with previous studies that demonstrated women with less education may have limited access to both antenatal education and empowerment, which affect their labor expectations and satisfaction [9]. Another study found that urban women are more satisfied than their rural counterparts because they frequently have greater access to better healthcare and experienced professionals [10]. Obstetric data indicated that patients with negative experiences of childbirth had elevated risks of emergency cesarean section, extended labor, and a reduced prevalence of spontaneous vaginal births. It aligns with the findings of investigative research that emphasizes that an unplanned surgical birth may produce a feeling of loss of control and trauma [11]. Long labor has also been known to elevate fatigue and distress, especially in the event of poor pain management [12]. The lower probabilities of spontaneous vaginal delivery within the negative group further support a study that highlighted the positive influence on psychological outcomes through perceived autonomy during childbirth [13].

The study results indicated that the psychological and hospital-related factors were the major contributors to childbirth experiences. The negative group of women was less likely to have a birth companion, pain relief, or supportive staff behavior experience. These findings demonstrated the significance of emotional and interpersonal support as revealed by previous studies [14,15]. However, the results found extensive labor to be a strong contributor to dissatisfaction, in contrast to research that found no correlation between labor duration and childbirth perception [16]. This can be explained by varying expectations and pain management across different settings. A correlation was found in an investigation between negative experiences of women in hospitals and longer hospitalization, which indicates complex deliveries or even slower emotional recovery [17,18].

Limitations of this study included the small, single-center sample size and the use of self-reported experiences that can lead to social desirability or recall bias. Furthermore, causal associations were not assessed due to the cross-sectional study design. Additionally, the previous birth experience, psychological state during birth, and cultural expectations were not evaluated, which can impact the perceptions of participants. In the future, further research needs to utilize longitudinal and multicenter study designs to investigate the long-term effects of the childbirth experience on mental health, satisfaction, and delivery preferences.

## Conclusions

This study identified the most significant socio-demographic, obstetric, and psychosocial factors related to adverse childbirth experiences. Women with lower education, living outside an urban area, experiencing long labor, undergoing urgent cesarean sections, lacking the presence of a birth companion, receiving weak pain management, and experiencing bad staff behavior were more likely to report dissatisfaction.

These observations confirm that both clinical practices and emotional aspects of care are critical determinants of maternal perceptions of childbirth experience and their overall satisfaction with the procedure.

## Appendices

**Table 6:** Self-Made Questionnaire to Assess the Determinants of Childbirth Experience and Delivery Mode Decisions Credits: Study Authors, Names (Sana Danish, Shama Chaudhary, Asma Ambareen, Maryum Sana). This is a questionnaire originally developed specifically for this study. No external sources were cited, and no permissions or license were required.

Questionnaire to Check Childbirth Experience							
A. Demographics Data							
Sr no.	Questions						
1	Age: ____ years						
2	Education: No formal ☐ / Primary ☐ / Secondary ☐ / Higher ☐						
3	Parity: Primiparous ☐ / Multiparous ☐						
4	Pregnancy type: Planned ☐ / Unplanned ☐						
5	Previous delivery mode: Vaginal ☐ / Cesarean ☐ / NA ☐						
6	Place of delivery: Public ☐ / Private ☐ / Other ☐						
B. Childbirth Experience							
Section	Sr No.	Statement	1 = Strongly Disagree	2	3	4	5 = Strongly Agree
Own Capacity	7	I was able to handle the pain during labour.	☐	☐	☐	☐	☐
	8	I felt strong and capable during childbirth.	☐	☐	☐	☐	☐
	9	I was able to manage my emotions during labour.	☐	☐	☐	☐	☐
Professional Support	10	The healthcare staff treated me with respect.	☐	☐	☐	☐	☐
	11	I received the information I needed during labour.	☐	☐	☐	☐	☐
	12	I felt supported by healthcare staff during childbirth.	☐	☐	☐	☐	☐
Perceived Safety	13	I felt safe during labour and delivery.	☐	☐	☐	☐	☐
	14	I trusted the healthcare team with my care.	☐	☐	☐	☐	☐
	15	My baby’s wellbeing was prioritized during delivery.	☐	☐	☐	☐	☐
Participation	16	I was involved in decisions about my labour and delivery.	☐	☐	☐	☐	☐
	17	My wishes and preferences were respected.	☐	☐	☐	☐	☐
	18	I felt I could influence what happened during childbirth.	☐	☐	☐	☐	☐
C. Negative Experience Determinants							
	19	I felt fear during childbirth.	☐	☐	☐	☐	☐
	20	I felt a lack of control over the process.	☐	☐	☐	☐	☐
	21	I experienced unnecessary medical interventions.	☐	☐	☐	☐	☐
	22	I felt ignored or not listened to by the staff.	☐	☐	☐	☐	☐
	23	I experienced trauma or distress during delivery.	☐	☐	☐	☐	☐
D. Mode of Delivery Decision							
	24	I am confident about my preferred mode of delivery in the future.	☐	☐	☐	☐	☐
	25	I feel uncertain about whether I should choose vaginal or cesarean delivery next time.	☐	☐	☐	☐	☐
	26	I feel I have enough information to make an informed choice.	☐	☐	☐	☐	☐
	27	I feel pressure from others (family, doctors) about the mode of delivery.	☐	☐	☐	☐	☐
	28	I feel supported in making my own decision about delivery mode.	☐	☐	☐	☐	☐
E. Future Delivery Preference							
	29	Based on my experience, I would prefer my next delivery to be: Vaginal ☐ / Cesarean ☐ / Undecided ☐					
